# Potential predictors for health-related quality of life in stroke patients undergoing inpatient rehabilitation

**DOI:** 10.1186/s12955-015-0314-5

**Published:** 2015-08-05

**Authors:** Chien-Min Chen, Chih-Chien Tsai, Chia-Ying Chung, Chia-Ling Chen, Katie PH Wu, Hsieh-Ching Chen

**Affiliations:** Department of Physical Medicine and Rehabilitation, Chang Gung Memorial Hospital, Chiayi, No.6, Sec. W., Jiapu Rd., Puzih City, Chiayi County 61363 Taiwan; School of Medicine, College of Medicine, Chang Gung University, Taoyuan, No.259, Wunhua 1st Rd., Kuei-Shan Dist. Taoyuan City, 33302 Taiwan; Department of Physical Medicine and Rehabilitation, Chang Gung Memorial Hospital, Linkou, No.5, Fushing St. , Kuei-Shan Dist. Taoyuan City, 33305 Taiwan; Graduate Institute of Early Intervention, College of Medicine, Chang Gung University, Taoyuan, No.259, Wunhua 1st Rd. , Kuei-Shan Dist. Taoyuan City, 33302 Taiwan; Department of Industrial Engineering & Management, National Taipei University of Technology, No.1, Sec. 3, Zhongxiao E. Rd., Daan Dist. Taipei City, 10608 Taiwan

**Keywords:** Stroke, Predictor, Health-Related Quality of Life, Rehabilitation

## Abstract

**Background:**

Improving HRQOL is the desired outcome for patients with stroke undergoing inpatient rehabilitation services. This study aimed to comprehensively identify the potential health-related quality of life (HRQOL) predictors in patients with stroke undergoing inpatient rehabilitation within the first year after stroke; thus far, such an investigation has not been conducted.

**Methods:**

We enrolled 119 patients (88 males, 31 females) with stroke, and examined 12 potential predictors: age, sex, stroke type, stroke side, duration after onset, cognition (Mini-Mental State Examination; MMSE), depression (Beck Depression Inventory-II), stroke severity (National Institutes of Health Stroke Scale; NIHSS), upper- and lower-extremity motor function scores of the Fugl–Meyer Assessment (FMA) scale, balance (Berg Balance Scale; BBS), and functional status (Functional Independence Measure). HRQOL was measured using Stroke Impact Scale (SIS) 3.0.

**Results:**

NIHSS score predicted the strength domain and total SIS score (41.5 % and 41.7 % of the variances, respectively). BBS score was a major predictor of mobility and participation/role domains (48.6 % and 10 % of the variances, respectively). MMSE score predicted the memory and communication domains (22.5 % and 36.3 % of the variances, respectively). Upper extremity score of the FMA scale predicted the daily living/instrumental activities of daily life and hand function domains (40.3 % and 20.6 % of the variances, respectively). Stroke side predicted the emotion domain (11.6 % of the variance).

**Conclusions:**

NIHSS, MMSE, BBS, FMA, and stroke side predicted most HRQOL domains. These findings suggest that different factors predicted various HRQOL domains in patients with stroke.

## Background

Stroke is a major health problem across the world. It has been long established that stroke has wide-ranging effects in physical, mental, and social life domains [1]. Health-related quality of life (HRQOL) refers to the health impacts on an individual’s functioning and on his or her perceived wellness in various life domains [2]. Stroke survivors have lower mean scores for physical health (−7.9 %), mental health (−4.1 %), and health utility (−6.9 %) of HRQOL than the nonstroke population [3].

According to the review of literature, age [4], sex [4], stroke type [5], stroke side [6], duration after onset [7], stroke severity [8], functional status [9], upper extremity (UE) motor function [10], balance function [11], lower extremity (LE) motor function [12], cognition function [8], and depression [13] have been identified as potential predictors of HRQOL outcomes in patients with stroke. The potential HRQOL predictors are varied among studies possibly because of different predictors and HRQOL domains investigated in each study. For example, age was identified as an HRQOL predictor for patients with stroke in Strum et al.’s study [4] but not in Fatoye et al.’s study [14]. To the best of our knowledge, only few studies [15, 16] have investigated a comprehensive list of HRQOL predictors in various domains in patients with stroke.

Recovery in stroke patients receiving rehabilitation primarily occurs in the first 3 months after stroke and continues in the following 3 months [17]. In addition, 6.5 % patients with stroke surviving for at least 1 year after stroke have been reported to show improvements in the 6–12 months after stroke [17]. Improving HRQOL is also the desired outcome for patients with stroke undergoing inpatient rehabilitation services. Mackenzie et al. [18] suggested that HRQOL predictors in patients with stroke admitted to rehabilitation hospitals during 0.5–3 months following stroke were the functional ability, physical, and psychological dimensions of HRQOL. Froes et al. [13] evaluated the factors affecting HRQOL [using Short Form-36 (SF-36)] in 64 patients (≥6 months after stroke) admitted to a rehabilitation program. They found that functional status affected physical functioning and physical role, and that depression could affect vitality, social function, emotional role, and mental health [13]. HRQOL after stroke varies considerably between different countries [19, 20]. This may be due to differences in the guidelines and strategies for post-stroke care and rehabilitation services. Compliance to post-acute-stroke rehabilitation guidelines could also be a quality-of-care indicator and affect HRQOL in patients with stroke undergoing inpatient rehabilitation [21]. Hopman et al. [22] reported that all 8 domains of the SF-36 improved in patients with stroke during inpatient rehabilitation; however, five domains declined at 6 months follow-up after discharge. Post-acute-stroke rehabilitation plays an important role in HRQOL. In Taiwan, the health care policy (National Health Insurance) allows most post-stroke patients to be admitted for inpatient rehabilitation several times within first year after a stroke. However, articles have comprehensively investigated HRQOL predictors for patients with stroke who were currently undergoing inpatient stroke rehabilitation during the first year after stroke are still lacking.

Many scales, including generic scales, such as SF-36 and EuroQol-5 Dimension Questionnaire (EQ-5D) [23–25], and stroke-specific [26] scales, have been used to assess HRQOL in patients with stroke. SF-36 was the most widely used generic HRQOL measurement [25]. However, the ceiling and floor effects that exist in some SF-36 domains [27] limit its use in the evaluation of patients with stroke. The Stroke Impact Scale (SIS) 3.0 is a comprehensive stroke-specific HRQOL measurement instrument [28]. SIS was developed to assess eight domains for mild-to-moderate stroke patients and it has been used in Taiwan [29, 30].

Identifying potential HRQOL predictors in patients with stroke will allow clinicians to predict HRQOL outcomes and plan early and appropriate treatment strategies. The research question was whether potential predictors for HRQOL differed among various domains in patients with stroke undergoing inpatient rehabilitation. After reviewing past articles, 12 potential predictors were selected for examination: age (at assessment), sex, stroke type (hemorrhagic or ischemic), stroke side (left or right), duration after onset, stroke severity, cognition function, depression, balance, UE and LE motor function, and functional status. We measured HRQOL with the SIS 3.0. We hypothesized that different predictors are associated with various HRQOL domains in patients undergoing inpatient stroke rehabilitation during the 1st year after stroke. The aim of this study was to reveal a comprehensive list of determinant factors for various HRQOL domains and total HRQOL score for patients receiving inpatient rehabilitation within 1 year after stroke.

## Methods

### Participants

Participants with stroke were recruited from the inpatient rehabilitation ward of a tertiary hospital. The diagnosis of stroke, lesion side, and stroke type were confirmed by a physiatrist by history evaluation, physical examination, brain imaging, and chart review. The physiatrist also determined a patient’s eligibility for the study. The inclusion criteria were (a) first-time unilateral cerebral stroke, (b) stroke onset ≤1 year, (c) admission to the rehabilitation ward, (d) ages 30–80 years, and (e) no active medical problems or physical conditions. The exclusion criteria were (a) brainstem or cerebellar stroke, (b) severe cognitive impairment, (c) severe aphasia, and (d) poor cooperation with assessments. Patients with brainstem or cerebellar stroke were excluded from the study because the symptoms differ from the typical hemiplegia or hemiparesis of cerebral stroke. Of the 258 patients with stroke initially assessed for eligibility, 121 were excluded because they did not meet the criteria and 18 declined to participate in the study. In total, 119 patients with stroke (88 males, 31 females) were included. The study protocol was approved by the Institutional Review Board for Human Studies at Chang Gung Memorial Hospital. All participants signed informed consent forms before enrollment.

### Assessment procedures

Two trained raters (a physical therapist and an occupational therapist) administered all measures. Raters reviewed the written instructions and were trained through repeated practice; a senior certified physical therapist or a senior certified occupational therapist assessed rater competence. All potential predictors were identified and HRQOL outcomes were measured for each study participant 2 to 3 weeks after admission for inpatient rehabilitation. All measurements for each participant were completed on the same day. The rehabilitation program offered services including both physical and occupational therapies. In addition, speech therapy was provided if required. Each of the daily physical, occupational, and speech therapy sessions usually lasted over 50 minutes on weekdays. Physical therapy included facilitation techniques, balance and transfer training, therapeutic exercise, strengthening exercise, and ambulation training. Occupational therapy involved motor task training, coordination training, hand function training, daily activities training, and visual perception training. Speech therapy consisted of auditory comprehension training, cognitive training, verbal production, oromotor coordination, augmentative communication, swallowing, and feeding training.

### Outcome measure of HRQOL

The SIS 3.0 was used for HRQOL measurement. SIS is a stroke-specific and self-reported instrument [31], consisting of 59 items designed for assessing 8 functional domains, including strength, memory, emotion, communication, activities of daily living (ADL)/instrumental activities of daily living (IADL), mobility, hand function, and participation/role. The internal consistency (Cronbach α: 0.94) and validity of SIS 3.0 was proved to be adequate. Participants responded to items in each domain using a 5-point rating scale. For strength items, 1 point meant “no strength at all” and 5 points meant “a lot of strength.” For memory, communication, ADL/IADL, mobility, and hand function items, 1 point meant “extremely difficult/cannot do at all” and 5 points meant “not difficult at all.” For emotion and participation/role items, 1 point meant “all of the time” and 5 points meant “none of the time.” Each domain of SIS has a range of 0–100 with higher scores indicated better QOL. Twenty-eight items in the four domains of strength, hand function, ADL/IADL, and mobility were combined to produce the composite physical domain.

### Potential predictors

The Mini-Mental State Examination (MMSE) [32] was used to measure cognition function, the Beck Depression Inventory (BDI)-II [33] was used to measure depression status, the National Institutes of Health Stroke Scale (NIHSS) [34] was used to measure stroke severity, the Berg Balance Scale (BBS) [35] was used to measure balance function, the Fugl–Meyer Assessment (FMA) [36]-UE motor function was used to measure UE motor function, the FMA-LE motor function was used to measure LE motor function, and the Functional Independence Measure (FIM) [37] was used to measure functional status.

#### MMSE

MMSE [32] is a brief 11-question test widely used for the evaluation of cognitive impairment. It measures orientation, registration, attention and calculation, recall, and language. The maximum total score is 30 points with lower total scores suggesting more severe cognitive impairment.

#### BDI-II

BDI-II [33] is a 21-item inventory that assesses the severity of depressive symptoms. Each item is rated on a 4-point scale range of 0–3. A score of 0 means “not feeling sadness at all” and 3 means “intolerable sadness or unhappiness.” The total score range on the BDI-II is 0–63.

#### NIHSS

NIHSS [34] is a 15-item assessment tool that provides a quantitative measure of stroke-related neurological deficit. NIHSS evaluates levels of consciousness, visual field loss, extraocular movement, facial muscle function, UE and LE motor strength, coordination, aphasia, dysarthria, sensory loss, extinction, and loss of attention. Total NIHSS scores range from 0 to 42, with higher scores meaning more severe neurological deficit.

#### BBS

BBS [35] is a 14-item test for evaluating static and dynamic balance ability. Score range of each item is 0–4; a score of 0 meant “unable to perform the task” and a score of 4 meant “able to independently perform the task without difficulty.” The final measure is the sum of all scores, with the lowest and highest possible total scores being 0 and 56, respectively.

#### FMA

FMA [36] is a stroke-specific, performance-based impairment index. It is designed to assess motor functioning, balance, sensation, and joint functioning in patients with post-stroke hemiplegia. Scoring is based on direct observation of performance. Scale items are scored on the basis of ability to complete the task using a 3-point scale where 0 = cannot perform, 1 = performs partially, and 2 = performs fully. FMA has a score range of 0–100 (66 UE, 34 LE), with 100 indicating normal motor performance.

#### FIM

The FIM [37] is an instrument designed for functional status and frequently used for patients with stroke. The FIM includes 18 items, each with a maximum score of 7 and a minimal score of 1. A score of 7 means complete independence and a score of 1 means complete dependence. Possible FIM total scores range from 18 to 126. Higher score means more independent functional status.

### Statistical analysis

Statistical analyses were performed with SPSS 18.0 for Windows. The dependent variables were the SIS 3.0. A two-step process determined whether a variable was considered a predictor of outcome measurements. First, a Pearson correlation coefficient (*r*) determined correlations between potential predictors and scores on the outcome measures (8 domains and total score). A *P* value of 0.05 was required for inclusion in the regression analysis. Potential predictors were used in a stepwise procedure to generate a linear regression model for outcome measurements. Adjusted *R*^2^, *P* values, and regression coefficients (β) were used to assess goodness-of-fit in the regression models. Regression diagnostics were also performed to check the multicollinearity among the predictors in the regression models.

## Results

Table 1 lists the demographic and clinical characteristics of the patients (mean age 54.89 years, standard deviation: 10.47 years).

**Table 1:** Demographic characteristics, clinical characteristics, and health-related quality of life in patients with stroke

	Value^a^
	Mean (SD) or number (%)
Demographic characteristics	
Age (years)	54.89 (10.47)
Sex	
Male	88 (73.9 %)
Female	31 (26.1 %)
Clinical characteristics	
Stroke type	
Hemorrhage	62 (52.1 %)
Infarction	57 (47.9 %)
Stroke side	
Left	59 (49.6 %)
Right	60 (50.4 %)
Duration after onset (months)	6.04 (3.24)
MMSE	26.39 (4.17)
BDI-II	14.65 (9.81)
NIHSS	4.31 (3.00)
BBS	38.45 (14.99)
FMA-UE	35.27 (21.86)
FMA-LE	31.59 (10.00)
FIM	88.25 (26.29)
SIS	
Strength	8.71 (3.01)
Memory	27.71 (6.03)
Emotion	28.54 (5.43)
Communication	30.37 (5.04)
ADL/IADL	29.26 (8.84)
Mobility	28.72 (9.74)
Hand function	9.92 (6.03)
Participation/Role	22.49 (9.02)
Total	185.72 (34.14)

^a^Continuous and categorical variables were displayed as mean (SD) and number (%), respectively

*MMSE* Mini-Mental State Examination; *BDI-II* Beck Depression Inventory-II; *NIHSS* National Institute of Health Stroke Scale; *BBS* Berg Balance Scale; *FMA* Fugl–Meyer Assessment; *UE* upper extremity; *LE* lower extremity; *FIM* Functional Independence Measure; *SIS* Stroke Impact Scale; *ADL* activities of daily living; *IADL* instrumental activities of daily living; *SD* standard deviation

### Correlation between potential predictors and HRQOL

Table 2 lists Pearson correlation coefficients of the 12 possible predictors and the scores on various QOL outcome measures. Age and sex were not associated with any domain of SIS. Stroke type was associated with communication and hand function (patients with infarction had better communication and hand function than patients with hemorrhage). Stroke side was associated with emotion, mobility, participation/role, and total SIS score (patients with left side lesion had better scores in these domains than those with right side lesion). Duration after onset was associated with strength (longer duration after onset leads to less strength). All clinical predictors were associated with most domains of SIS.

**Table 2:** Relationships between the potential predictors and SIS domains in patients with stroke

Candidate predictors	Pearson's *r*								
	SIS domains								
	Strength	Memory	Emotion	Communication	ADL/IADL	Mobility	Hand function	Participation/Role	Total
Demographic									
Age	0.006	0.096	−0.099	0.123	−0.033	−0.135	0.082	0.067	0.005
Sex	0.056	−0.071	−0.052	−0.017	0.028	−0.036	0.053	−0.030	−0.020
Stroke type	0.002	0.079	0.023	0.218^*^	0.063	0.092	0.221^*^	0.043	0.143
Stroke side	−0.134	−0.084	−0.352^*^	0.056	−0.133	−0.188^*^	−0.025	−0.257^*^	−0.234^*^
Duration after onset	−0.215^*^	−0.163	−0.066	−0.152	0.028	0.051	−0.009	0.012	−0.057
Clinical									
MMSE	0.423^*^	0.481^*^	0.109	0.607^*^	0.433^*^	0.470^*^	0.272^*^	0.124	0.556^*^
BDI-II	−0.158	−0.298^*^	−0.293^*^	−0.202^*^	−0.200^*^	−0.234^*^	−0.086	−0.095	−0.302^*^
NIHSS	−0.648^*^	−0.332^*^	−0.196^*^	−0.425^*^	−0.570^*^	−0.540^*^	−0.382^*^	−0.267^*^	−0.649^*^
BBS	0.544^*^	0.192^*^	0.214^*^	0.245^*^	0.600^*^	0.701^*^	0.312^*^	0.328^*^	0.649^*^
FMA-UE	0.599^*^	0.338^*^	0.109	0.416^*^	0.639^*^	0.554^*^	0.461^*^	0.189^*^	0.646^*^
FMA-LE	0.536^*^	0.371^*^	0.109	0.447^*^	0.584^*^	0.633^*^	0.387^*^	0.167	0.640^*^
FIM	0.507^*^	0.363^*^	0.165	0.386^*^	0.580^*^	0.600^*^	0.403^*^	0.235^*^	0.647^*^

^*^ *P*–values < 0.05

*MMSE* Mini-Mental State Examination; *BDI-II* Beck Depression Inventory-II; *NIHSS* National Institute of Health Stroke Scale; *BBS* Berg Balance Scale; *FMA* Fugl–Meyer Assessment; *UE* upper extremity; *LE* lower extremity; *FIM* Functional Independence Measure; *SIS* Stroke Impact Scale; *ADL* activities of daily living; *IADL* instrumental activities of daily living

### Potential predictors of predicting HRQOL by regression analysis

Table 3 presents the results of the stepwise multiple regression analyses. NIHSS score, FIM score, and duration after onset were predictors in the strength domain of the SIS (adjusted *R*^2^ = 0.540) (*P* < 0.001). MMSE and BDI-II scores were predictors in the memory domain of the SIS (adjusted *R*^2^ = 0.290) (*P* < 0.001). Stroke side and BDI-II score were predictors in the emotion domain of the SIS (adjusted *R*^2^ = 0.169) (*P* < 0.001). MMSE score, stroke type, and FMA-UE score were predictors in the communication domain of the SIS (adjusted *R*^2^ = 0.413) (*P* < 0.001). FMA-UE, FIM, and BBS scores were positive predictors in the ADL/IADL domain of the SIS (adjusted *R*^2^ = 0.532) (*P* < 0.001). BBS, FIM, FMA-LE, and BDI-II scores were predictors in the mobility domain of the SIS (adjusted *R*^2^ = 0.605) (*P* < 0.001). FMA-UE and FIM scores were positive predictors of hand function in the SIS (adjusted *R*^2^ = 0.240) (*P* < 0.001). BBS score was a positive predictor in the participation/role domain of the SIS (adjusted *R*^2^ = 0.100) (*P* < 0.001). NIHSS, FIM, FMA-LE, BDI-II, MMSE, and BBS scores were predictors of the total SIS score (adjusted *R*^2^ = 0.677) (*P* < 0.001).

**Table 3:** The predictors for the health-related quality of life in patients with stroke by stepwise multiple regression analyses

Dependent variables	Independent variables	Unstandardized coefficient (β)	standardized coefficient (β)	Lower limit of 95 % CI	Upper limit of 95 % CI	Adjusted *R* ^*2*^	*F*	*P*
SIS domains								
Strength							47.094	<0.001
	Constant	8.741		6.983	10.500			
	NIHSS	−0.460	−0.514	−0.580	−0.340	0.415		
	FIM	0.038	0.333	0.023	0.054	0.491		
	Duration after onset	−0.212	−0.228	−0.328	−0.096	0.540		
Memory							25.154	<0.001
	Constant	13.927		8.187	19.668			
	MMSE	0.626	0.464	0.418	0.834	0.225		
	BDI-II	−0.157	−0.267	−0.247	−0.066	0.290		
Emotion							12.986	<0.001
	Constant	35.711		32.692	38.729			
	Stroke side	−3.412	−0.315	−0.292	−5.232	0.116		
	BDI-II	−0.130	−0.246	−0.292	−0.219	0.169		
Communication							28.709	<0.001
	Constant	11.233		6.551	15.914			
	MMSE	0.598	0.530	0.426	0.771	0.363		
	Stroke type	1.612	0.160	0.184	3.040	0.394		
	FMA-UE	0.039	0.173	0.004	0.075	0.413		
ADL/IADL							45.772	<0.001
	Constant	11.335		7.333	15.338			
	FMA-UE	0.146	0.365	0.084	0.208	0.403		
	FIM	0.088	0.261	0.036	0.139	0.492		
	BBS	0.147	0.266	0.059	0.234	0.532		
Mobility							46.223	<0.001
	Constant	7.809		3.172	12.446			
	BBS	0.262	0.431	0.171	0.354	0.486		
	FIM	0.088	0.238	0.036	0.141	0.560		
	FMA-LE	0.185	0.227	0.061	0.310	0.592		
	BDI-II	−0.123	−0.130	−0.234	−0.013	0.605		
Hand function							19.644	<0.001
	Constant	2.269		−1.095	5.634			
	FMA-UE	0.094	0.347	0.045	0.144	0.206		
	FIM	0.053	0.231	0.011	0.095	0.240		
Participation/Role							14.152	<0.001
	Constant	15.420		11.388	19.452			
	BBS	0.185	0.328	0.088	0.283	0.100		
Total							42.236	<0.001
	Constant	112.671		83.405	141.937			
	NIHSS	−2.434	−0.240	−3.930	−0.938	0.417		
	FIM	0.392	0.302	0.223	0.561	0.602		
	FMA-LE	0.402	0.140	−0.013	0.817	0.633		
	BDI-II	−0.555	−0.167	−0.911	−0.199	0.650		
	MMSE	1.310	0.171	0.346	2.274	0.668		
	BBS	0.352	0.165	0.017	0.686	0.677		

*MMSE* Mini-Mental State Examination; *BDI-II* Beck Depression Inventory-II; *NIHSS* National Institute of Health Stroke Scale; *FMA* Fugl–Meyer Assessment; *UE* upper extremity; *LE* lower extremity; *BBS* Berg Balance Scale; *FIM* Functional Independence Measure; *SIS* Stroke Impact Scale; *ADL* activities of daily living; *IADL* instrumental activities of daily living; *CI* confidence interval

The nine final regression equations were as follows:

$$ \mathrm{Strength}=8.741-(0.460)\ \mathrm{NIHSS}+(0.038)\ \mathrm{F}\mathrm{I}\mathrm{M}-(0.212)\ \mathrm{duration}\ \mathrm{after}\ \mathrm{onset} $$

$$ \mathrm{Memory}=13.927+(0.626)\ \mathrm{MMSE}-(0.157)\ \mathrm{B}\mathrm{D}\mathrm{I}\hbox{-} \mathrm{I}\mathrm{I} $$

$$ \mathrm{Emotion}=35.711-(3.412)\ \mathrm{stroke}\ \mathrm{side}\ \left(\mathrm{left}:\ 1;\ \mathrm{right}:\ 2\right)-(0.130)\ \mathrm{B}\mathrm{D}\mathrm{I}\hbox{-} \mathrm{I}\mathrm{I} $$

$$ \mathrm{Communication}=11.233+(0.598)\ \mathrm{M}\mathrm{M}\mathrm{SE}+(1.162)\ \mathrm{stroke}\ \mathrm{type}\ \left(\mathrm{hemorrhage}:\ 1;\ \mathrm{infarction}:\ 2\right)+(0.039)\ \mathrm{F}\mathrm{M}\mathrm{A}\hbox{-} \mathrm{U}\mathrm{E} $$

$$ \mathrm{A}\mathrm{D}\mathrm{L}/\mathrm{IADL}=11.335+(0.146)\ \mathrm{F}\mathrm{M}\mathrm{A}\hbox{-} \mathrm{U}\mathrm{E}+(0.088)\ \mathrm{F}\mathrm{I}\mathrm{M}+(0.147)\ \mathrm{B}\mathrm{B}\mathrm{S} $$

$$ \mathrm{Mobility}=7.809+(0.262)\ \mathrm{B}\mathrm{B}\mathrm{S}+(0.088)\ \mathrm{F}\mathrm{I}\mathrm{M}+(0.185)\ \mathrm{F}\mathrm{M}\mathrm{A}\hbox{-} \mathrm{L}\mathrm{E}-(0.123)\ \mathrm{B}\mathrm{D}\mathrm{I}\hbox{-} \mathrm{I}\mathrm{I} $$

$$ \mathrm{Hand}\ \mathrm{function}=2.269+(0.094)\ \mathrm{F}\mathrm{M}\mathrm{A}\hbox{-} \mathrm{U}\mathrm{E}+(0.053)\ \mathrm{F}\mathrm{I}\mathrm{M} $$

$$ \mathrm{Participation}/\mathrm{Role}=15.420+(0.185)\ \mathrm{B}\mathrm{B}\mathrm{S} $$

$$ \mathrm{Total}=112.671-(2.434)\ \mathrm{NIHSS}+(0.392)\ \mathrm{F}\mathrm{I}\mathrm{M}+(0.402)\ \mathrm{F}\mathrm{M}\mathrm{A}\hbox{-} \mathrm{L}\mathrm{E}-(0.555)\ \mathrm{B}\mathrm{D}\mathrm{I}\hbox{-} \mathrm{I}\mathrm{I}+(1.310)\ \mathrm{M}\mathrm{M}\mathrm{S}\mathrm{E}+(0.352)\ \mathrm{B}\mathrm{B}\mathrm{S} $$

## Discussion

This is the first study on the potential predictors of various HRQOL domains in patients with stroke receiving inpatient rehabilitation during the first year after stroke. BBS score was a major factor in predicting the mobility and participation/role. FMA-UE scores were a major factor in predicting hand function and ADL/IADL domains, respectively. MMSE score was the main factor in predicting the memory and communication domains. NIHSS score was a major factor in predicting the strength domain. Stroke side was a major factor in predicting the emotion domain. Our findings suggested that different factors predicted the various HRQOL domains in patients with stroke. The BBS, FMA-UE, MMSE, NIHSS scores, and stroke side predicted most HRQOL domains. This information may allow clinicians to plan specific treatment strategies for patients with stroke.

Our findings are partially in agreement with previous studies [13, 16, 18]. Using SIS as the HRQOL measure, Carod-Artal et al. [16] surveyed the predictors for HRQOL in patients with stroke at outpatient neurology and stroke rehabilitation clinics. They found that the NIHSS and functional status (Barthel Index) were the main predictors for the strength domain, the MMSE and the hospital anxiety and depression scales were the main predictors for the memory domain, the hospital anxiety and depression scale were the main predictors for the emotional domain, MMSE was a predictor for the communication domain, functional status (Barthel Index) and the hospital anxiety and depression scale were predictors for the mobility domain, and functional status (Barthel Index) was a predictor for the ADL/IADL and hand function domains. Froes et al. [13] and Mackenzie et al. [18] surveyed predictors for HRQOL in patients with stroke currently undergoing inpatient rehabilitation. Froes et al. [13] showed that FIM and time after stroke onset could predict the physical functioning domain, and that the BDI-II was a major predictor for vitality, emotional role, and mental health domains of SF-36. Using the sickness impact profile (SIP) as the HRQOL measure, Mackenzie et al. found that baseline and 2-week functional status (Modified Barthel Index) could be a predictor for total SIP score at 2 weeks and 3 months individually after admission [18]. Some inconsistent results among studies may be due to the patient characteristics (stroke types and lesion sides), rehabilitation programs (e.g. inpatient or ambulatory rehabilitation), onset time after stroke, selected HRQOL assessment tools, selected predictors, etc.

In this study, BBS predicted variances in several domains of HRQOL, such as participation/role and mobility domains, explaining 10 %–48.6 % of the variances. However, UE and LE motor functions were not identified as major predictors of variances in these domains. This may be because balance control is more important for mobility and participation in social activities. Ng illustrated that balance ability was associated with functional mobility in stroke survivors [38]. Patterson et al.’s study asserted that balance function was more important than paretic limb strength for long distance walking in stroke patients with severe gait deficit [39]. In Kollen et al.’s study [40], improvement in standing balance control was more important than improvement in LE strength or synergism in achieving walking ability improvements in patients with stroke. Schmid et al. illustrated that balance function was independently associated with participation in patients with chronic stroke [41]. These findings may suggest balance training should be included in the rehabilitation for enhancing the mobility and participation of HRQOL domains.

Good cognition can predict better memory and communication. We found that MMSE was an important predictor for memory and communication accounting for 22.5 %–36.3 % of the variances. Our result was similar to Carod-Artal et al.’s [16] study that evaluated the determinants of HRQOL (using SIS 3.0) in 260 patients with stroke and found that MMSE score was a predictor of the memory and communication domains. However, in another article, cognition also affected physical function and other domains of HRQOL. In Patel et al.’s study [42], cognitive impairment (MMSE score <24) was identified as a predictor for lower physical component score and mental component score on SF-36. Jeong et al.’s article [8] also illustrated that MMSE score was independently associated with physical factors, psychological factors, and environmental context of HRQOL. The different results may be due to different predictors and HRQOL measurements among the various studies.

Extremity motor function predicted various HRQOL domains. UE motor function predicted hand function and ADL/IADL of HRQOL, whereas LE motor function was a predictor for the mobility. Nicolas-Larsen et al.’s study [5] found decreased UE motor function was a predictor for poorer composite physical domain (combined strength, ADL/IADL, mobility, and hand function) score on the SIS 3.0 in patients 3–9 months after stroke. Lin et al. [43] found that FMA-UE was a predictor for hand function, ADL/IADL, and participation/role domains in patients 6 months after stroke. Franceschini et al. [12] found that incomplete LE motor recovery was a predictor for a lower HRQOL in the mobility domain, and incomplete UE motor recovery was a predictor for a lower HRQOL in usual activities and self-care domains. These findings may suggest that treatment strategies should include UE and LE training to promote HRQOL in various domains.

BDI-II predicted HRQOL to a lesser extent than other clinical factors. In our study, BDI-II was a predictor for the memory, emotion and mobility domains, but only accounted for 1.3 %–6.5 % of the variances. Though the variance is low, depression could affect patient motivation for participating in rehabilitation programs and mobility tasks to a certain degree. Froes et al.’s study [13] used BDI to measure the effect of depression on HRQOL (SF-36) and found that BDI was not only a significant factor for mental health, emotional role, social functioning, but also for the vitality scale. Kwok et al. [44] evaluated HRQOL of 268 patients with stroke who had received inpatient rehabilitation for 3 weeks during the acute stage. They followed up these patients at 3, 6, and 12 months after stroke and found that more severe depression was associated with decreased physical, psychological, social interaction, and environmental HRQOL domains [44]. Depression status seems to affect HRQOL of a patient with stroke in both the psychological domain and certain non-psychological domains. These findings may suggest that psychosocial support and treatment of depression may be needed to enhance some HRQOL domains.

Stroke severity predicted the strength domain and the total SIS score in patients with stroke. In this study, NIHSS predicted strength domain, explaining 41.5 % of the variance and predicted total SIS score, explaining 41.7 % of the variance. In Carod-Artal et al.’s article [16], NIHSS > 6 was identified as a predictor for composite physical domain and multiple domains, including strength, ADL/IADL, participation/role, mobility, and hand function for stroke survivors. Sturm et al.’s [4] articles showed that the NIHSS score 7 days after stroke could be used to predict total HRQOL score at 2 years after stroke. It seems stroke severity is a robust factor in predicting total HRQOL of patients with stroke, particularly in physical domains.

In our study, stroke side predicted 11.6 % of the variance in the emotion domain, which was greater than was explained by depression. Right hemisphere stroke has a lower HRQOL in the emotion domain than left hemisphere stroke in our study. Rachpukdee et al. [45] also illustrated right hemisphere stroke was a negative predictor for the mental health domain of HRQOL at one month post-stroke. Although right cerebral hemisphere is dominant for sense of emotion [46], some previous articles did not show the same result as ours. In Morris et al. article [10], left hemisphere stroke was significantly associated with, but did not predict poorer emotional reactions. Hopman et al. [22] illustrated the left hemisphere stroke predicted worse emotional functioning than those with right hemisphere stroke at their admission for rehabilitation. However, no difference of stroke side on emotional functioning existed in these patients at their discharge and 6-month follow-up [22]. Although some studies have shown that lesion side had impacts on the emotional domain of HRQOL, other studies have not. One previous study [6] showed that lesion side only had impacts on the body care, movement, and communication, but not on the emotional domain of HRQOL. Another article [9] showed that lesion side did not influence the HRQOL score values in first-ever stroke patients. Therefore, the effect of stroke side on emotion seems to be inconclusive and warrants further study. Lincoln et al. [47] showed that the severity of anxiety and depression markedly increased between 6 months and 5 years after stroke. Therefore, anxiety should also be included in further studies in predicting HRQOL for patients with stroke at both the acute stage and follow-up stages.

The major limitations in this study were participant characteristics and selection of potential predictors. Only participants with unilateral cerebral stroke who were receiving inpatient rehabilitation were included. In addition, other confounding factors influencing HRQOL, such as environmental factors [48, 49], family support [50], psychosocial factors [11, 48], or rehabilitation services [51], were not included in this study. For example, ongoing home-based [51] or ambulatory rehabilitation services (physical therapy, occupational therapy, or speech therapy) are probably important confounding factors influencing HRQOL.

## Conclusion

In conclusion, the NIHSS was the major predictor for strength and global HRQOL in patients with stroke. Different factor combinations predicted various HRQOL domains in patients with stroke. BBS, MMSE, FMA-UE, NIHSS scores, and stroke side predicted most HRQOL domains. FIM, FMA-LE, BDI-II scores, duration after onset, and stroke type may influence some HRQOL domains. These findings suggest that these predictors may be useful for the early identification of patients who have the most potential to acquire HRQOL. More importantly, these results may allow clinicians to plan appropriate rehabilitation intervention and enhance HRQOL in patients with stroke by implementing cognitive training (e.g., training involving attention, memory, intellectual execution, visual perception, auditory comprehension, and verbal expression), balance training (e.g., static and dynamic balance training involving sitting balance, standing balance, single leg balance, sit-to-stand or stand-to-sit training, and transferring training), and motor functional training (e.g., hand dexterity, object manipulation, visual motor coordination, locomotion, agility, and body coordination). Future studies should focus on longitudinal follow-up and include other potential predictors for HRQOL in patients with stroke.

## Abbreviations

HRQOL: Health-related quality of life
SF-36: Short Form-36
SIS: Stroke Impact Scale
ADL: Activities of daily living
IADL: Instrumental activities of daily living
MMSE: Mini-Mental State Examination
BDI: Beck Depression Inventory
NIHSS: National Institutes of Health Stroke Scale
BBS: Berg Balance Scale
FMA: Fugl–Meyer Assessment
UE: Upper extremity
LE: Lower extremity
FIM: Functional Independence Measure
